# Discovery of a Novel ERp57 Inhibitor as Antiplatelet Agent from Danshen* (Salvia miltiorrhiza)*

**DOI:** 10.1155/2018/9387568

**Published:** 2018-04-24

**Authors:** Jia Zou, Yang Chen, Maggie Pui Man Hoi, Jun Li, Tao Wang, Ying Zhang, Yu Feng, Jianli Gao, Simon Ming Yuen Lee, Guozhen Cui

**Affiliations:** ^1^Zhuhai Key Laboratory of Basic and Applied Research in Chinese Medicine, Department of Bioengineering, Zunyi Medical University, Zhuhai Campus, Zhuhai, China; ^2^State Key Laboratory of Quality Research in Chinese Medicine and Institute of Chinese Medical Sciences, University of Macau, Macau; ^3^Academy of Traditional Chinese Medicine, Zhejiang Chinese Medical University, Hangzhou, Zhejiang 310053, China

## Abstract

Danshen* (Salvia miltiorrhiza)* is a well-known herb in Traditional Chinese Medicine (TCM) for treating cardiovascular diseases, but the underlying mechanism remains to be fully elucidated. Here, we showed that Danshen and its active ingredient rosmarinic acid exhibited antiplatelet effects through the inhibition of ERp57, a member of protein disulfide isomerase (PDI) with potential roles in platelet aggregation. Danshen extract (DSE) exhibited potent inhibitory effects on the platelet aggregation induced by arachidonic acid- (AA-) induced platelet aggregation and the enzymatic activity of ERp57. Rosmarinic acid was identified by virtual screening and molecular docking as one of the hit compounds for ERp57. In line with this, rosmarinic acid displayed significant inhibitory effect on ERp57 activity and inhibited AA-induced platelet aggregation. Taken together, we demonstrated for the first time that DSE and rosmarinic acid displayed inhibitory effects on the catalytic activity of ERp57, providing evidence of the regulatory role of ERp57 underlying the antiplatelet effects of Danshen.

## 1. Introduction

Cardiovascular diseases (CVDs) are the leading cause of death, accounting for almost one-third of global deaths every year [1]. In China, the mortality rate due to CVDs is the highest among other diseases and the incidence of CVDs is increasing continuously [2]. Abnormal platelet function and blood coagulation have been implicated to play significant roles in the pathogenesis and complications of CVDs, and the signaling pathways of platelet activation and aggregation are primary targets for treatment [3]. Clopidogrel and aspirin are the current antiplatelet therapies for the treatment and management of patients with CVDs for inhibiting platelet aggregation, thrombus formation, and subsequent risk of thromboembolism events [4], but they have limitations and side effects such as increased risk of hemorrhage and resistance [5].

ERp57 is a thiol oxidoreductase enzyme in the family of protein disulfide isomerase (PDI) which mediates protein folding and redox signaling by promoting the formation of disulfide bonding [6]. Recent studies with ERp57 gene-deficient mice and anti-ERp57 antibody have shown that ERp57 might have important roles in platelet aggregation and thrombus formation* in vivo* in addition to its regulations in apoptosis and antitumor responses [7, 8]. In line with this, we previously demonstrated that danshensu derivative ADTM, (R)-(3,5,6-trimethylpyrazinyl) methyl-2-acetoxy-3-(3,4-diacetoxyphenyl) propanoate, could bind to ERp57, inhibit its catalytic activity, and display protective effects by attenuating acute ischemic myocardial infarct through inhibiting thrombus formation in rat [9–13]. Therefore, we hypothesize that agents with inhibitory effects on ERp57 may be potential promising antiplatelet therapy for cerebrovascular diseases. Similar to other members in the PDI family, ERp57 consists of four thioredoxin-like domains ((a), (b), (b′), and (a′)). The catalytic domains (a) and (a′) each contain two CGHC active site motifs [14], and mutational study of ERp57 showed that the second active site (C406-G407-H408-C409) is critical for platelet aggregation [7]. Structure-based virtual screening is a powerful tool for the identification of potential ligands for protein targets; however, no ERp57 inhibitor has been reported using virtual screening yet.

Danshen* (Salvia miltiorrhiza)* is well-known for its effect of promoting blood circulation and is widely used in China for treating CVDs, and its usage has been increasing in North America and Europe in recent years [15]. Danshen contains water-soluble compounds (e.g., salvianolic acids) and lipid-soluble compounds (e.g., tanshinones) [16]. Recent studies reported that danshensu is the major active component for the vasorelaxant and antioxidant effects of Danshen [16]. Other studies showed that salvianolic acid A inhibited platelet aggregation by interacting with phosphoinositide 3-kinase [17] and salvianolic acid B inhibited platelet aggregation induced by adenosine diphosphate (ADP) and collagen [18, 19]. Danshensu and salvianolic acid B were reported to be responsible for protective effects against hepatic toxicity induced by paracetamol, an analgesic and antipyretic drug [20]. Despite the substantial progress made in the study of the underlying mechanisms of Danshen, major active components of Danshen responsible for promoting blood circulation are still not identified. In our present study, we evaluated the effects of Danshen extract (DSE) on platelet function and ERp57 activity by light transmission aggregometry and insulin reduction assay, respectively. We further employed structure-based virtual screening to identify the major active components of Danshen which bind to ERp57, and the hit compounds were validated by insulin reduction assay and antiplatelet assay.

## 2. Materials and Methods

### 2.1. Animal

New Zealand white rabbits were bought from Guangdong Medical Laboratory Animal Center. Animals were housed under standard conditions, fed with normal forage, and given water ad libitum. The animal experiments were approved by the Animal Care and Experimentation Committee of Zunyi Medical University in March 2011 and were performed in accordance with the approved guidelines.

### 2.2. Chemicals and Reagents

Dithiothreitol, aspirin, clopidogrel bisulfate, and rosmarinic acid (purity > 97% by HPLC) were obtained from Aladdin Company (Shanghai, China). Daucosterol (purity > 98% by HPLC) was obtained from Baoji Chenguang Biotechnology Company (Baoji, China). Insulin and collagen were obtained from Sigma-Aldrich (St Louis, MO, USA). AA and ADP were purchased from Helena Laboratories (Beaumont, TX, USA). Danshen was obtained from a distributor of medicinal plants in June 2016, and the medicinal materials were identified by Dr. Yang Chen (Biological Engineering Department, Zunyi Medical University, Zhuhai, China). The voucher specimen (number ZMC10) was deposited in Zhuhai Key Laboratory of Fundamental and Applied Research in Traditional Chinese Medicine.

### 2.3. DSE Preparation

The DSE was prepared as follows: 100 g of Danshen soaked in 400 mL 75% ethanol-water solution at 25°C for 12 h. The solution was filtered through Whatman no. 1 filter paper. The solid residue was then heated in water at 60°C for 1.5 h. The extracted solutions were all concentrated to dryness and stored in a refrigerator (4°C) for subsequent analysis.

### 2.4. Insulin Reduction Assay* In Vitro*

The cDNA for ERp57 was kindly provided by Dr. Wu (Soochow University, Suzhou, China) and recombinant ERp57 was expressed using* Escherichia coli* expression system and purified as previously described [8]. The turbidity of insulin was measured to reflect the ERp57 activity [21, 22]. The assays were conducted with 96-well plates. After adding 10 *μ*L ERp57 (0.04 mg/mL) and 10 *μ*L vehicle (0.1% DMSO), DSE, rosmarinic acid, or daucosterol solution to 50 *μ*L insulin (1 mg/mL), 10 *μ*L dithiothreitol (DTT, 1.5 mM) was incubated in the former reaction solutions, and the final volume was 80 *μ*L in each well. The insulin needs to be freshly prepared in assay buffer containing 100 *μ*M KH_2_PO_4_, 2 mM EDTA (PH: 7.4). The change of turbidity was monitored at 630 nm using microplate reader (Thermo Fisher Scientific, Massachusetts, USA) at 20°C for 84 min. ERp57 enzyme inhibition was determined by following formula [23]: enzyme inhibition (%) = [1 − (OD_[compound + ERp57 + DTT]_ − OD_[DTT]_)/(OD_[ERp57 + DTT]_ − OD_[DTT]_)]*∗*100%.

### 2.5. Molecular Docking and Virtual Screening

Molecular Operating Environment (MOE, version 2016. 08, Chemical Computing Group Inc., Montreal, QC, Canada) software was used to calculate the binding energy between ERp57 and small molecule compounds of Danshen. X-ray 3D structure was downloaded from the PDB database (PDBID: 3F8U). After protein structure preparation concerning removing excess water molecules, adding hydrogen and structural energy minimization, second active site (C406-G407-H408-C409) in ERp57 was used as an active pocket for screening small molecular compound in Danshen. 179 current known compounds of natural small molecules in Danshen were used to establish a small molecular library. The structures of small molecules were converted into a 3D structure and then were imported into the MOE software for analysis and processing. Docking experiments were carried out using the default parameters of MOE (Placement: Triangle Matcher, Rescoring: London dG, Refinement: Forcefield).

### 2.6. Platelet Isolation and Platelet Aggregation

Rabbit blood was collected in 3.8% sodium citrate vacuum anticoagulant tube and centrifuged at 100*g* for 15 min to obtain Platelet-rich plasma (PRP). Platelet aggregation was carried out as our previously described with minor modifications [10]. PRP was incubated with vehicle (0.1% DMSO), rosmarinic acid (1, 3, 10, 30, and 100 *μ*M), DSE (15, 50, 150, 450, and 1350 *μ*g/mL), aspirin (100 *μ*M), or clopidogrel bisulfate (100 *μ*M) for 5 min at 37°C. Platelet aggregation was induced by 0.24 mM AA, 9 *μ*M ADP, or 10 *μ*g/mL collagen and monitored using platelet aggregometer (Helena Laboratories Corp., Beaumont, TX, USA). The rate of maximum (max) aggregation was defined by the highest level of platelet aggregation within 5 min. Inhibition of platelet aggregation was calculated by the following formula: inhibition rate = [(rate of max aggregation in the control group − rate of max aggregation in compound treated group)/rate of max aggregation in control group] *∗* 100%. All IC_50_ values were determined from the corresponding concentration inhibition curve by SPSS software (12.0 version).

### 2.7. Statistical Analysis

Data were expressed as mean  ±  SD of three independent experiments. Statistical significance was completed using one-way ANOVA followed by Tukey's multiple comparison tests. All the analyses were performed with SPSS version 12.0 statistical software. The value of* P* < 0.05 was considered statistically significant.

## 3. Results

### 3.1. DSE Inhibited Platelet Aggregation* In Vitro*

**Figure 1:**
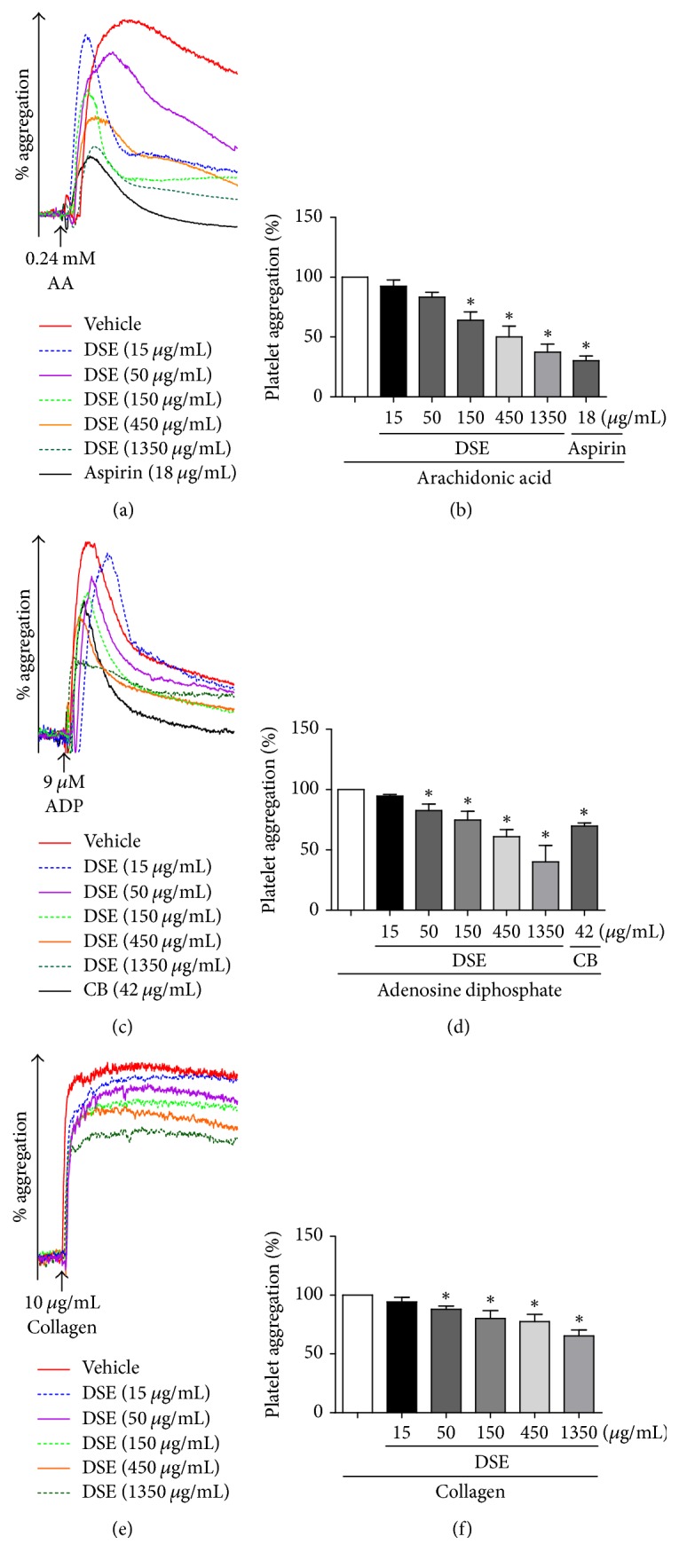
Inhibitory effect of DSE on platelet aggregation* in vitro*. Platelets were pretreated for 5 min with various concentrations of DSE (15, 50, 150, 450, and 1350 *μ*g/mL), aspirin (18 *μ*g/mL), clopidogrel bisulfate (42 *μ*g/mL), or vehicle, followed by simulation with 0.24 mM AA (a, b), 9 *μ*M ADP (c, d), or 10 *μ*g/mL collagen (e, f). The platelet maximum aggregation rate of revulsant treatment group was normalized to 100%. ^*∗*^*P* < 0.05 compared with revulsant treatment group. Data are expressed as mean ± SD, *n* ≥ 3/group. CB: clopidogrel bisulfate.

We first examined the toxicity of DSE on platelets. The platelets were treated with different concentrations (15, 50, 150, 450, and 1350 *μ*g/mL) of DSE for 20 min followed by LDH assay. As shown in Supplementary Figure 1, DSE up to 1350 *μ*g/mL did not demonstrate toxicity. In order to investigate the effect of DSE on platelet activation, platelet aggregation induced by revulsants, AA, ADP, or collagen was measured with light transmission aggregometry in PRP. As expected, aspirin and clopidogrel bisulfate, served as positive controls, significantly inhibited platelet aggregation induced by AA or ADP, respectively. DSE (15, 50, 150, 450, and 1350 *μ*g/mL) also inhibited the platelet aggregation induced by the three revulsants in a concentration-dependent manner (*P* < 0.05, Figure 1). The half maximal inhibitory concentration (IC_50_) of DSE against AA or ADP-induced platelet aggregation was 508.79 ± 78.80 *μ*g/mL and 775.85 ± 123.29 *μ*g/mL, respectively. It is noted that DSE was more effective in antiplatelet activity against AA-induced platelet aggregation than that of ADP and collagen. In line with these studies, we found that ADP significantly increased ATP release, which was reversed by DSE in a concentration-dependent manner (Supplementary Figure 2A).

### 3.2. DSE Inhibited Reductive Activity of ERp57* In Vitro*

**Figure 2:**
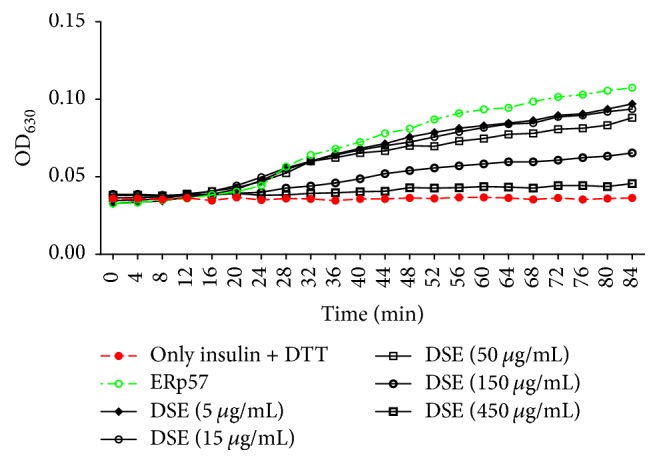
Inhibitory effect of different concentrations of DSE on the activity of ERp57. The ERp57 was incubated with different concentrations of DSE (5–450 *μ*g/mL) for 84 min, and its catalytic activity was determined by insulin turbidity assay.

ERp57 emerges as a novel promising target for antiplatelet therapy while the underlying mechanisms involved in the antithrombotic activity of Danshen have long remained elusive. To investigate whether ERp57 is a target of DSE, the effect of DSE on ERp57 activity was determined by insulin reduction assay. As shown in Figure 2, DSE inhibited the activity of ERp57 in a concentration- and time-dependent manner. The IC_50_ of DSE on ERp57 activity was 87.00 ± 12.15 *μ*g/mL.

### 3.3. Virtual Screening Results of Small Molecules in Danshen

**Figure 3:**
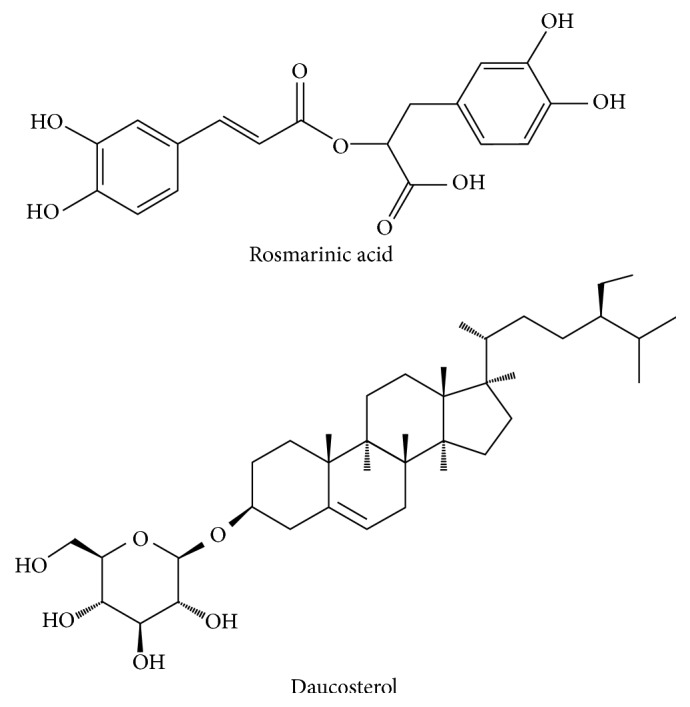
Structures of rosmarinic acid and daucosterol selected from virtual screening for pharmacological validation.

**Figure 4:**
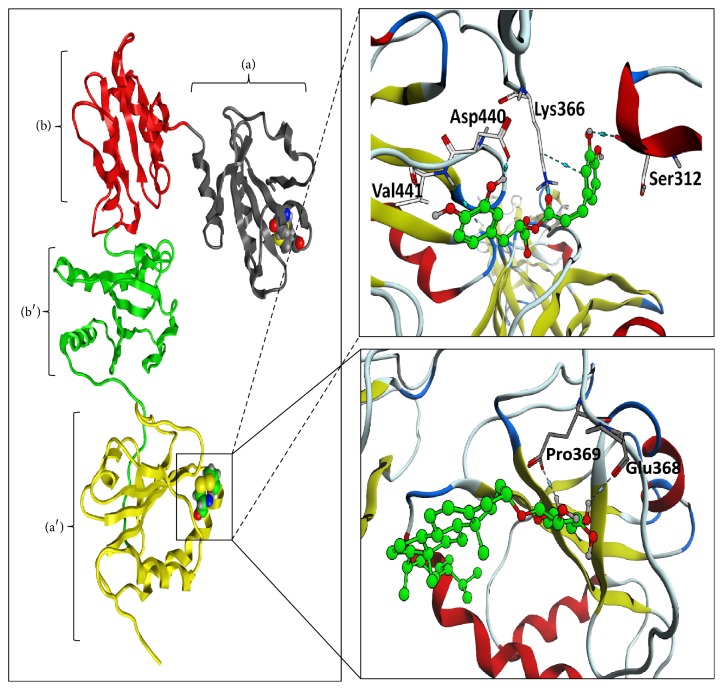
Docked poses of rosmarinic acid and daucosterol with ERp57. The left diagram represents the three-dimensional (3D) model of ERp57. The space-filling model in domains (a) and (a′) indicates the catalytic residues CGHC on the left. The enlarged figures of the right side show the interaction between ERp57 and rosmarinic acid (top right) or daucosterol (bottom right). The green mode of ball and stick represents rosmarinic acid or daucosterol and the crystal structure of ERp57 domain with each monomer presented in ribbon diagram. Hydrogen bonding is shown as light blue dashed line.

The X-ray structure of ERp57 (PDBID: 3F8U) was used for molecular docking. The search area for virtual screening was restricted to the second active site (C406-G407-H408-C409) of ERp57. A small molecule compound library was established with 179 small molecular compounds in Danshen and screened in silico. Rosmarinic acid and daucosterol were chosen for further study (Figure 3). The binding free energy of ERp57 with rosmarinic acid and daucosterol was −20.4 kcal/mol and −17.9 kcal/mol, respectively. The result of the three-dimensional (3D) molecular docking view for interaction showed that rosmarinic acid formed hydrogen bonds to Ser312, Lys366, Asp440, and Val441 of ERp57, while daucosterol formed hydrogen bonds to Glu368 and Pro369 of the protein (Figure 4).

### 3.4. Hit Compounds Validation by Insulin Reduction Assay

**Figure 5:**
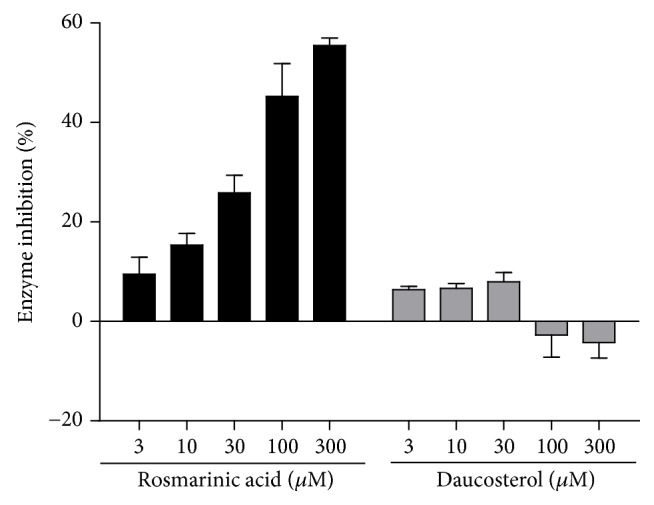
Rosmarinic acid inhibited the catalytic activity of ERp57. Effects of rosmarinic acid and daucosterol at 3, 10, 30, 100, and 300 *μ*M on ERp57 activity, as determined by insulin reduction assay. Rosmarinic acid, but not daucosterol, inhibited the activity of ERp57 in a concentration-dependent manner.

Hit compounds rosmarinic acid and daucosterol were validated by insulin reduction assay to evaluate their capacity to inhibit ERp57 catalytic activity. Rosmarinic acid inhibited the activity of ERp57 in a concentration-dependent manner while daucosterol did not show any inhibitory effect (Figure 5). The IC_50_ of rosmarinic acid on ERp57 activity was 176.82 ± 11.74 *μ*M.

### 3.5. Rosmarinic Acid Inhibited Platelet Aggregation* In Vitro*

**Figure 6:**
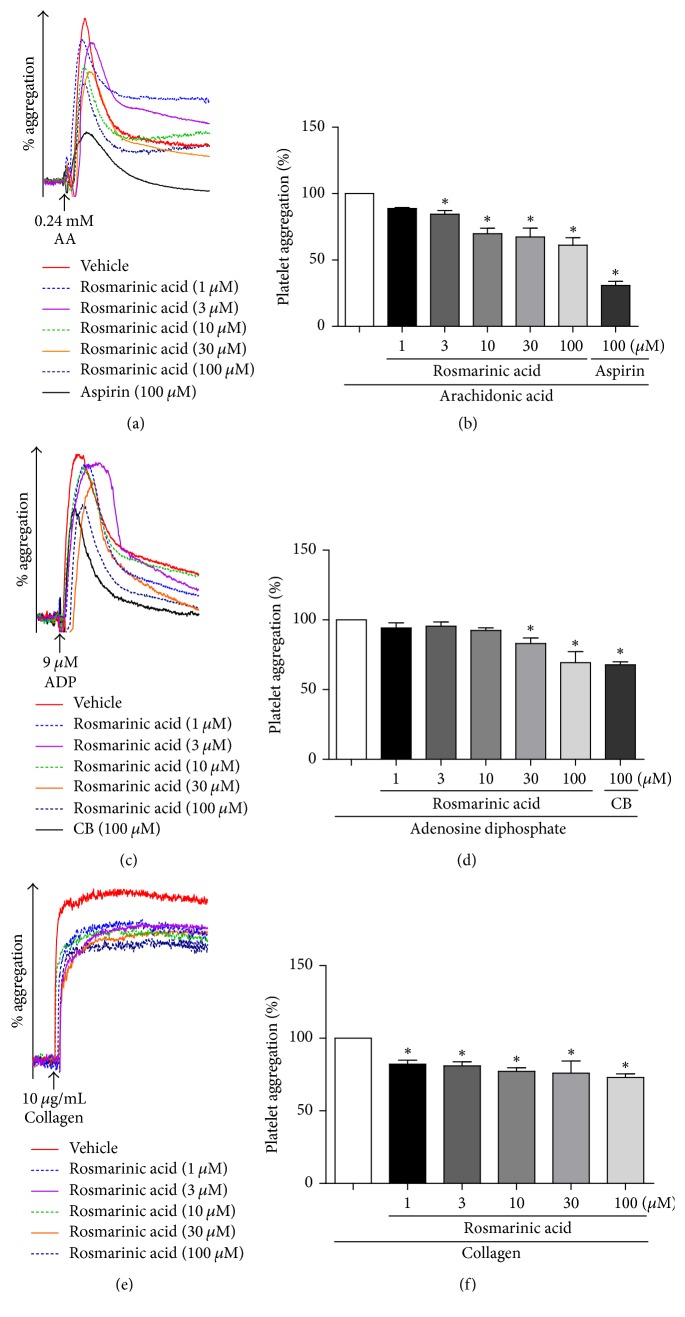
Inhibitory effect of rosmarinic acid on platelet aggregation. Platelets were pretreated for 5 min with various concentrations of rosmarinic acid (1, 3, 10, 30, and 100 *μ*M), aspirin (100 *μ*M), clopidogrel bisulfate (100 *μ*M), or vehicle. The platelets were stimulated with 0.24 mM AA (a, b), 9 *μ*M ADP (c, d) or 10 *μ*g/mL collagen (e, f). The platelet maximum aggregation rate of revulsant treatment group was normalized to 100%. ^*∗*^*P* < 0.05 compared with revulsant treatment group. Data are expressed as means ± SD, *n* ≥ 3/group. CB: clopidogrel bisulfate.

We further used various revulsants (AA, ADP, or collagen) to induce platelet activation and evaluated the antiplatelet effects of rosmarinic acid and daucosterol. The results demonstrated that rosmarinic acid, but not daucosterol, significantly inhibited the platelet aggregation induced by AA, ADP, or collagen (*P* < 0.05, Figure 6). Notably, there was no significant difference between 100 *μ*M rosmarinic acid and 100 *μ*M positive drug (clopidogrel bisulfate) treatment group in the platelet aggregation, which suggested that the inhibitory effect of 100 *μ*M rosmarinic acid on ADP-induced platelet aggregation was similar to that of 100 *μ*M clopidogrel bisulfate, a first-line antiplatelet drug in the treatment of unstable angina. Consistent with the antiplatelet activity of DSE, rosmarinic acid was more effective in antiplatelet activity against AA-induced platelet aggregation than that of ADP or collagen. Moreover, rosmarinic acid (1–100 *μ*M) markedly reduced ADP-induced ATP release (Supplementary Figure 2B). In contrast, daucosterol had no inhibitory effect against the three revulsants-induced platelet aggregation (Supplementary Figure 4).

## 4. Discussion

In the present study, we aimed to identify ERp57 inhibitors as potential antiplatelet agents from the active chemical components of Danshen by using structure-based virtual screening and experimental bioassays. DSE was prepared by ethanolic and hot water extraction, and the antiplatelet activity of DSE was evaluated. DSE exhibited significant inhibitory effects on the platelet aggregation induced by arachidonic acid (AA), ADP, or collagen (Figure 1). We further examined the inhibitory effect of DSE on ERp57 catalytic activity by insulin reduction assay. DSE inhibited the activity of ERp57 in a concentration- and time-dependent manner (Figure 2). Data analysis of the structure-based virtual screening against ERp57 protein active site identified two compounds as promising ERp57 ligands, rosmarinic acid, and daucosterol, from the components of Danshen. Chemical analysis of DSE by LC-MS showed that rosmarinic acid was present in DSE (Supplementary Figure 3). We further demonstrated that rosmarinic acid, but not daucosterol, significantly inhibited the activity of ERp57 as well as platelet aggregation (Figure 6).

Rosmarinic acid was a water-soluble phenolic compound mainly found in plants in the Boraginaceae family, such as Danshen and rosemary. Rosmarinic acid was reported to display multiple biological effects including antioxidant, anti-inflammatory, antitumor, and anxiolytic activity [24]. Previous studies showed that rosmarinic acid downregulated indoleamine 2,3-dioxygenase expression by inhibiting cyclooxygenase and was correlated with the induction of immune tolerance [25]. In addition, rosmarinic acid inhibited 4-aminobutyrate transaminase, which is a therapeutic target in depression [26]. More recently, rosmarinic acid was identified in the aqueous extract of Danshen by platelet/cell membrane chromatography combined with UPLC-MS/MS, but rosmarinic acid (50 *μ*M) did not exhibit inhibitory effect against platelet activation induced by thrombin, ADP, or U4669 [3]. In line with this, we observed that rosmarinic acid (1, 3, 10, 30, and 100 *μ*M) significantly inhibited the platelet aggregation induced by AA or collagen.

Recent studies provided accumulating evidence that ERp57 has important roles in the regulation of platelet function and aggregation and is a potential target for antiplatelet therapy [7, 8, 27]. Thiol isomerase enzymes PDI and ERp57 are present in platelets, and they share high homology in structure. It has been shown that PDI inhibitors, such as quercetin-3-rutinoside, inhibited platelet aggregation and thrombosis [22]. In another study, ERp57 antibody inhibited the expression of integrin *α*IIb*β*3 and platelet aggregation in mice [8]. It is proposed that ERp57 might decrease platelet activity by inhibiting the calcium signals that regulate integrin *α*IIb*β*3 and fibrinogen [28]. However, until now the crystal structure of ERp57 is not yet available, thus impeding the progress of drug discovery with virtual screening targeting at ERp57. In this study, by performing structure-based virtual screening with the compound library of Danshen against the ERp57 active site protein pocket, we identified rosmarinic acid as a lead candidate for ERp57 inhibitor. Molecular docking analysis indicated that rosmarinic acid bound to ERp57 in the catalytic domain through hydrogen bonds. These results provided essential information for the further design of specific inhibitors for ERp57 as antiplatelet agents.

## 5. Conclusions

Our results showed that DSE significantly inhibited platelet aggregation possibly by inhibiting ERp57, and rosmarinic acid was identified by structure-based virtual screening as one major active compound. Our study provided insights into the underlying mechanisms that might explain the blood circulation promoting effects of Danshen.

## Supplementary Materials

Supplementary Materials Supplementary Figure 1: effect of DSE on LDH release from platelets. The washed platelets were preincubated with 0.1% DMSO (vehicle control) or DSE (15, 50, 150, 450, and 1350 *μ*g/mL) for 20 min at 37°C and were centrifuged and then supernatant was collected, according to the LDH assay kit's manufacturers' protocol to measurement. The LDH release of 0.1% DMSO treatment group was normalized to 100%. Data are expressed. Supplementary Figure 2: inhibitory effect of DSE or rosmarinic acid on ATP release induced by ADP. Platelets were pretreated for 5 min with various concentrations of vehicle, DSE (15, 50, 150, 450, and 1350 *μ*g/mL), rosmarinic acid (1, 3, 10, 30, and 100 *μ*M), or clopidogrel bisulfate (100 *μ*M), followed by the simulation with 9 *μ*M ADP. ATP release from platelets was evaluated by using a luciferase-based system. ^*∗*^*P* < 0.05 compared with ADP group. Data were expressed as mean ± SD, *n* ≥ 3/group. CB: clopidogrel bisulfate. Supplementary Figure 3: negative ion electrospray tandem mass spectra of rosmarinic acid. Supplementary Figure 4: effect of daucosterol on platelet aggregation* in vitro*. Platelets were pretreated without or with various concentrations of daucosterol (1, 10, and 100 *μ*M), aspirin (100 *μ*M, positive control), clopidogrel bisulfate (100 *μ*M, positive control), or vehicle for 5 min at 37°C. The platelets were further stimulated with 0.24 mM AA (A, B), 9 *μ*M ADP (C, D), or 10 *μ*g/mL collagen (E, F). The platelet maximum aggregation rate of revulsant treatment group was normalized to 100%. ^*∗*^*P* < 0.05 compared with revulsant treatment group. Data are expressed as mean ± SD, *n* ≥ 3/group. CB: clopidogrel bisulfate.
