# Supplementary material for: Discovery of a Novel ERp57 Inhibitor as Antiplatelet Agent from Danshen (Salvia miltiorrhiza)
Source: Evid Based Complement Alternat Med. 2018 Apr 24;2018:9387568. doi: 10.1155/2018/9387568 (PMC5941821; doi:10.1155/2018/9387568)
Supplement: Supplementary Materials — Supplementary Figure 1: effect of DSE on LDH release from platelets. The washed platelets were preincubated with 0.1% DMSO (vehicle control) or DSE (15, 50, 150, 450, and 1350 μg/mL) for 20 min at 37°C and were centrifuged and then supernatant was collected, according to the LDH assay kit's manufacturers' protocol to measurement. The LDH release of 0.1% DMSO treatment group was normalized to 100%. Data are expressed. Supplementary Figure 2: inhibitory effect of DSE or rosmarinic acid on ATP release induced by ADP. Platelets were pretreated for 5 min with various concentrations of vehicle, DSE (15, 50, 150, 450, and 1350 μg/mL), rosmarinic acid (1, 3, 10, 30, and 100 μM), or clopidogrel bisulfate (100 μM), followed by the simulation with 9 μM ADP. ATP release from platelets was evaluated by using a luciferase-based system. ∗P < 0.05 compared with ADP group. Data were expressed as mean ± SD, n ≥ 3/group. CB: clopidogrel bisulfate. Supplementary Figure 3: negative ion electrospray tandem mass spectra of rosmarinic acid. Supplementary Figure 4: effect of daucosterol on platelet aggregation in vitro. Platelets were pretreated without or with various concentrations of daucosterol (1, 10, and 100 μM), aspirin (100 μM, positive control), clopidogrel bisulfate (100 μM, positive control), or vehicle for 5 min at 37°C. The platelets were further stimulated with 0.24 mM AA (A, B), 9 μM ADP (C, D), or 10 μg/mL collagen (E, F). The platelet maximum aggregation rate of revulsant treatment group was normalized to 100%. ∗P < 0.05 compared with revulsant treatment group. Data are expressed as mean ± SD, n ≥ 3/group. CB: clopidogrel bisulfate. [file 9387568.f1.docx]

**Discovery of a novel ERp57 inhibitor as antiplatelet agent from Danshen** **(*Salvia miltiorrhiza*)**

Jia Zou^a^, Yang Chen^a^, Maggie Pui Man Hoi^b^, Jun Li^a^, Tao Wang^a^, Ying Zhang^a^, Yu Feng^b^, Jianli Gao^c^, Simon Ming Yuen Lee^b^, Guozhen Cui^a^

^a^Zhuhai key laboratory of basic and applied research in Chinese medicine, Department of Bioengineering, Zhuhai Campus of Zunyi Medical University, Zhuhai, ^b^State Key Laboratory of Quality Research in Chinese Medicine and Institute of Chinese Medical Sciences, University of Macau, Macao and ^c^Academy of Traditional Chinese Medicine，Zhejiang Chinese Medical University, Hangzhou, Zhejiang 310053, China

**Correspondence**

Yang Chen and Guozhen Cui, Zhuhai key laboratory of basic and applied research in Chinese medicine, Department of Bioengineering, Zhuhai Campus of Zunyi Medical University, Zhuhai, China. E-mail: chenyang197732@hotmail.com, cgzum@hotmail.com

**Supplementary methods and data**

**Supplementary methods**

**LC-MS analysis of Danshen extract (DSE)**

LC-MS date was obtained with a LC-MS/MS system. The system was consisted of an agilent technoloies 1260 infinity HPLC systerm and an agilent technologies 6130 quadrupole LC/MS. HPLC conditions: agilent proshell 120 EC-C18 column (3.0×150 mm, 2.7 μM) at 30^o^C with a flow rate of 0.43 mL/min. The mobile phase consisted of 0.1% formic acid-water (A) and acetonitrile (B). The gradient program was as follows: 0-20 min, 17-25% B; 20-22 min, 25-70% B; 22-30 min, 70-78% B; 30-31 min, 78-90% B; 31-38 min, 90% B; 38-40 min, 90-17% B. The injection volume was 10 μL. The concentration of sample was 1 mg/mL in 50% methanol-water (V/V) and the effluent monitored at 286 and 270 nm by a DAD detection.

**Preparation of washed platelets**

Rat blood was collected in 3.8% sodium citrate vacuum anticoagulant tube and centrifuged at 100 g for 15 min to obtain Platelet-rich plasma (PRP). The PRP was centrifuged at 1000 g for 10 min at 37°C. An then platelet pellets were suspended in Tyrode’s solution (pH 7.4) with the following compositions: 137 mM NaCl, 0.3 mM Na_2_HPO_4_, 2 mM KCl, 12 mM NaHCO_3_, 10 mM HEPES, 5.5 mM glucose, 1 mM MgCl_2_, 1 mM CaCl_2_, 0.3% bovine serum albumin (BSA). The washed platelets were suspended in the Tyrode’s solution and adjusted to 3.6 x 10^8^ platelets/mL [[1](#_ENREF_1), [2](#_ENREF_2)].

**Measurement of lactate dehydrogenase (LDH)**

Various concentrations of DSE (15, 50, 150, 450, 1350 μg/mL) or 0.1% DMSO was added to the washed platelets (3.6×10^8^ cells/mL) for 20 min at 37^o^C. Then the washed platelets were centrifuged at 1700 g, 10 min and collected supernatant. The assays were conducted with 96-well plates and according to the manufacturer’s protocol to measurement of LDH release. The levels of LDH were measured at 450 nm using microplate reader (Thermo Fisher Scientific, Massachusetts, USA).

**ATP release assay**

Platelets were pre-incubated for 5 min at 37^o^C with or without various concentrations of DSE, rosmarinic acid or clopidogrel bisulfate, then stimulated with 9 μM ADP for 5 min. ATP levels were measured using ATP assay kit (Beyotime, Shanghai, China) according to the manufacturer's instructions. Results were normalized to the ADP group, the ATP release of which was considered as 100% assay as described previously [[3](#_ENREF_3), [4](#_ENREF_4)].

**Platelet isolation and platelet aggregation**

Rabbit blood was collected in 3.8% sodium citrate vacuum anticoagulant tube and centrifuged at 100 g for 15 min to obtain Platelet-rich plasma (PRP). Platelet aggregation was carried out as our previously described with minor modifications [[5](#_ENREF_5)]. PRP was incubated with various concentrations of Daucosterol (1, 10, 100 μM) or 100 μM aspirin, Clopidogrel bisulfate for 5 min at 37^o^C. Platelet aggregation was induced by 10 μg/mL collagen, 0.24 mM AA or 9 μM ADP, respectively. And then it was monitored using a platelet aggregometer (Helena Laboratories Corp., Beaumont, TX, USA). Rate of maximum aggregation was defined by the highest level of platelet aggregation within 5 min. Inhibition of platelet aggregation was calculated by the following formula: inhibition rate = [(rate of max aggregation in control group-rate of max aggregation in compound treated group)/rate of max aggregation in control group]*100%.

**Supplementary data**

**Effect of DSE on LDH release from platelet**

The platelets were pre-incubated with 0.1% DMSO (vehicle) or various concentrations of DSE for 20 min. The results demonstrated that DSE (15, 50, 150, 450, 1350 μg/mL) did not significantly increase the LDH release compared with vehicle group (*P* > 0.05, Supplementary Fig. 1). This result indicated that DSE (15, 50, 150, 450, 1350 μg/mL) treatment for 20 min did not induce cytotoxic effects on platelets.


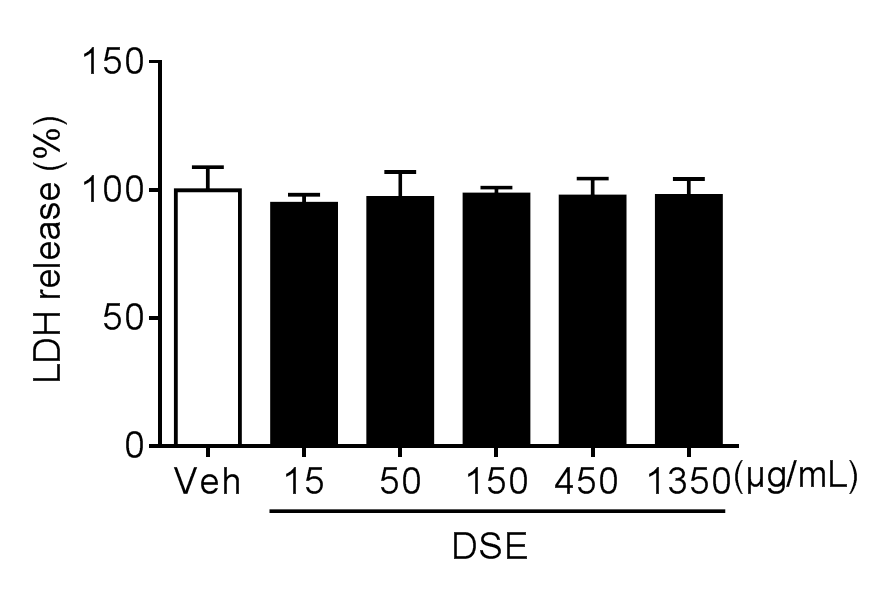


**Supplementary Figure 1** Effect of DSE on LDH release from platelets. The washed platelets were pre-incubated with 0.1% DMSO (vehicle control) or DSE (15, 50, 150, 450, 1350 μg/mL) for 20 min at 37^o^C, centrifugation and collected Supernatant, then according to the LDH assay kit’s manufacturer’s protocol to measurement. The LDH release of 0.1% DMSO treatment group was normalized to 100%. Data are expressed as means ± SD, n ≥ 3/group.

**DSE and rosmarinic acid inhibited ATP release**

Platelet granule release plays an important role in the initial of platelet aggregation. Here, experiment on ATP release, which was measured by the microplate reader, was carried out whether DSE and rosmarinic acid had an effect on the granule release-induced by ADP. As shown in Supplementary Fig. 2, 9 μM ADP significantly induced ATP release, which was obviously reversed by DSE, rosmarinic acid or clopidogrel bisulfate (*P* < 0.05). These results indicated that DSE or rosmarinic acid inhibited ADP-induced platelet activation via the blockade of granule release.


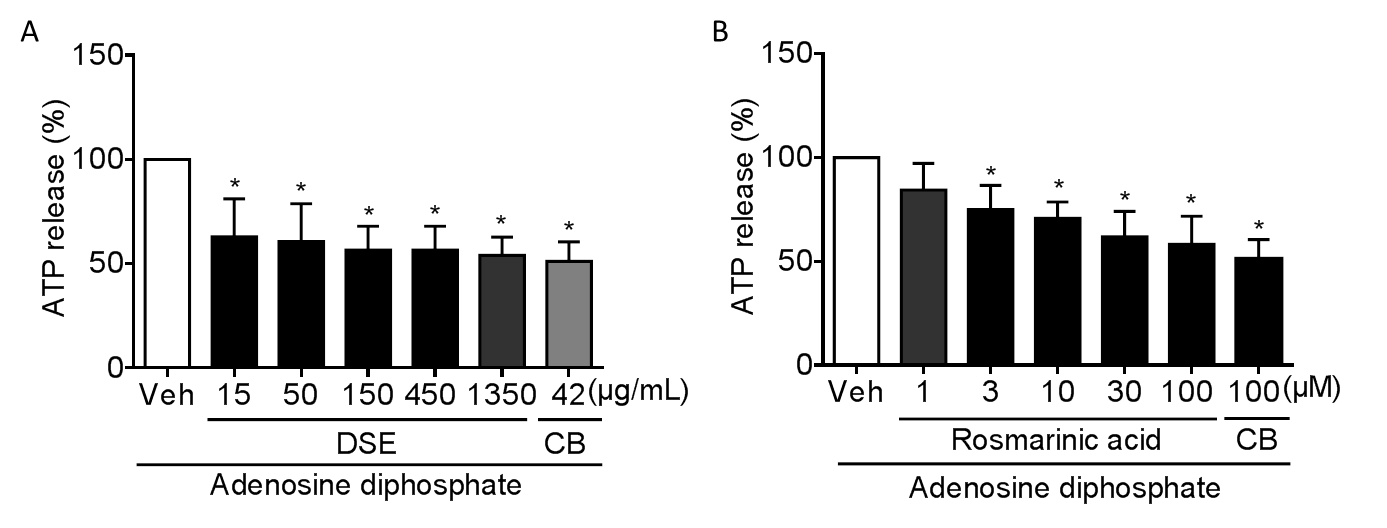


**Supplementary Figure 2** Inhibitory effect of DSE or rosmarinic acid on ATP release-induced by ADP. Platelets were pretreated for 5 min with various concentrations of vehicle, DSE (15, 50, 150, 450, 1350 μg/mL), rosmarinic acid (1, 3, 10, 30, 100 μM) or clopidogrel bisulfate (100 μM), followed by the simulation with 9 μM ADP. ATP release from platelets was evaluated by using a luciferase-based system. **P* < 0.05 compared with ADP group. Data were expressed as mean ± SD, n ≥ 3/group. CB: clopidogrel bisulfate.

**Identification the rosmarinic acid in DSE**

The major chemical components of DSE were characterized by liquid chromatography tandem-mass spectrometry (LC-MS) analysis in negative mode. As shown in supplementary Fig. 3, the molecular ion peak at M/Z 359.1, in agreement with the molecular formula of rosmarinic acid and consistent with previous [[6](#_ENREF_6)]. This result indicated that rosmarinic acid was contained in the DSE.


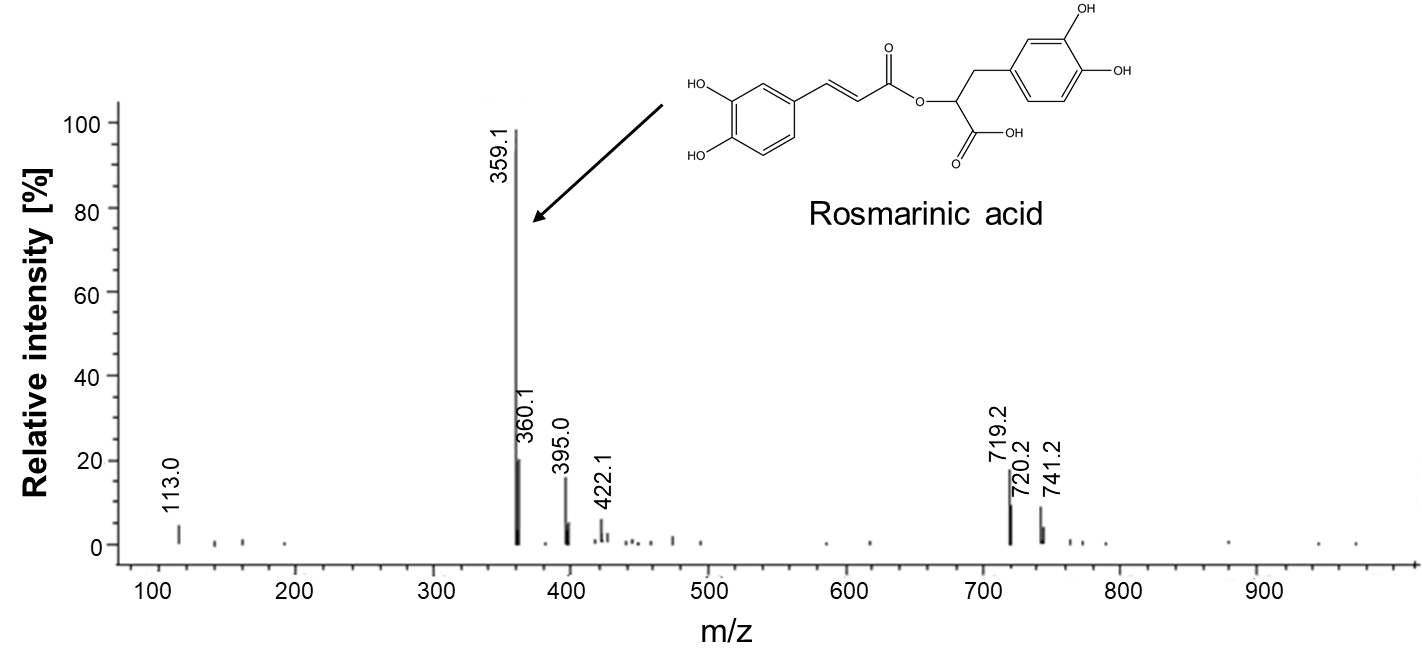


**Supplementary Figure 3** Negative ion electrospray tandem mass spectra^-^ of rosmarinic acid.

**Daucosterol did not inhibit platelet aggregation *in vitro***

We further used various revulsants (collagen, ADP or AA) to induce platelet activation and evaluated the effect of daucosterol. The results demonstrated that different concentrations of daucosterol (1, 10, 100 μM) had no inhibitory effect against ADP, AA or collagen-induced platelet aggregation (*P* > 0.05, Supplementary Fig. 4).


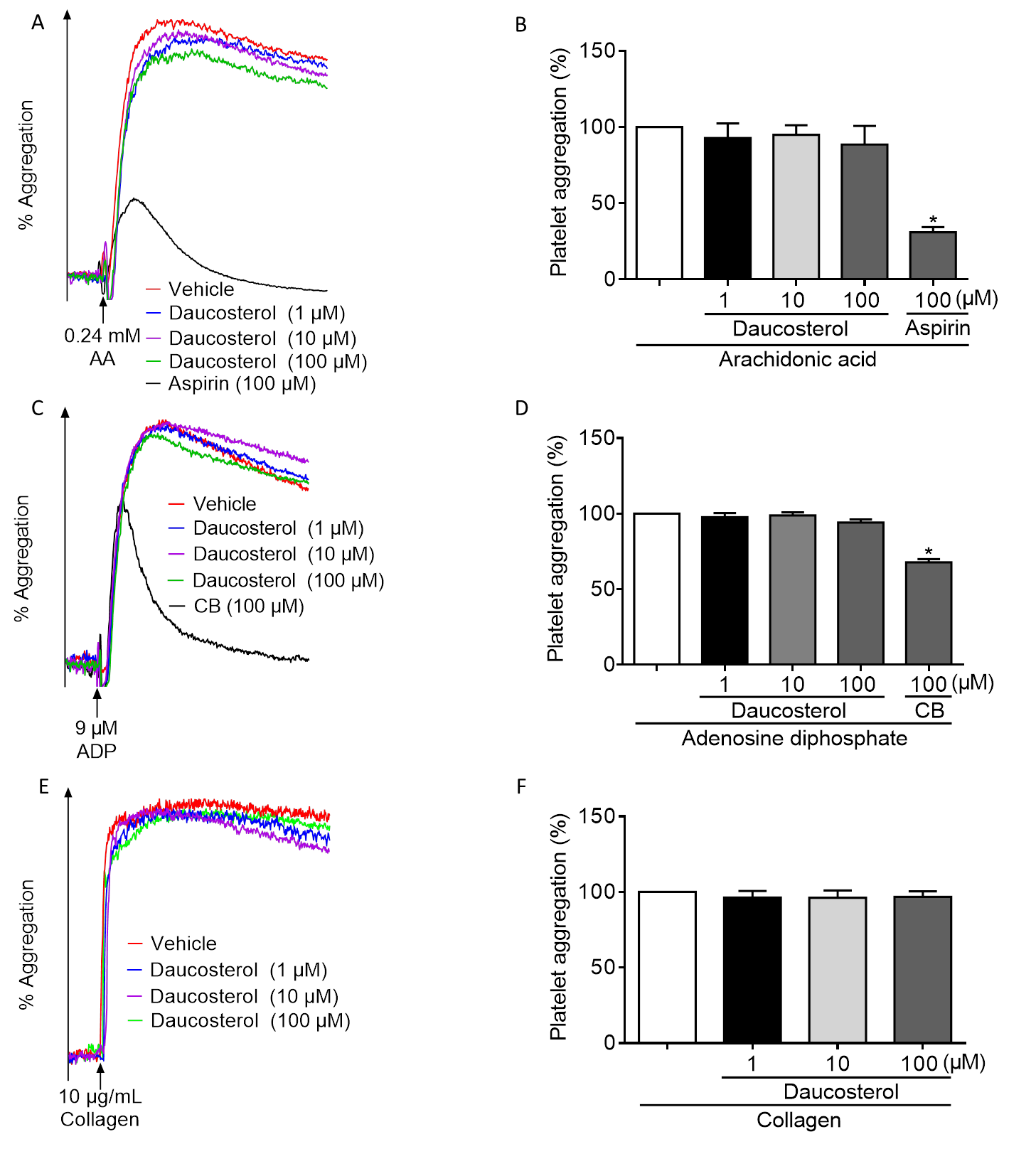


**Supplementary Figure 4** Effect of daucosterol on platelet aggregation *in vitro*. Platelets were pretreated without or with various concentrations of daucosterol (1, 10, 100 μM), aspirin (100 μM, positive control), clopidogrel bisulfate (100 μM, positive control) or vehicle for 5 min at 37°C. The platelets were further stimulated with 0.24 mM AA (A, B), 9 μM ADP (C, D) or 10 μg/mL collagen (E, F). The platelet maximum aggregation rate of revulsant treatment group was normalized to 100%. **P* < 0.05 compared with revulsant treatment group. Data are expressed as mean ± SD, n ≥ 3/group. CB: clopidogrel bisulfate.

**References**

1. L.M. Lien, K.H. Lin, L.T. Huang, et al. "Licochalcone A Prevents Platelet Activation and Thrombus Formation through the Inhibition of PLCgamma2-PKC, Akt, and MAPK Pathways," *Int J Mol Sci, vol.* 18, no. 7.

2. C.H. Hsia, M. Velusamy, J.R. Sheu, et al. "A novel ruthenium (II)-derived organometallic compound, TQ-6, potently inhibits platelet aggregation: Ex vivo and in vivo studies," *Sci Rep, vol.* 7, no. 1, p. 9556.

3. Y. Lu, Q. Li, Y.Y. Liu, et al. "Inhibitory effect of caffeic acid on ADP-induced thrombus formation and platelet activation involves mitogen-activated protein kinases," *Sci Rep, vol.* 5, p. 13824.

4. S. Zhang, J. Wang, S. Chen, et al. "Effects of Suilysin on Streptococcus suis-Induced Platelet Aggregation," *Front Cell Infect Microbiol, vol.* 6, p. 128.

5. G. Cui, L. Shan, L. Guo, et al. "Novel anti-thrombotic agent for modulation of protein disulfide isomerase family member ERp57 for prophylactic therapy," *Sci Rep, vol.* 5, p. 10353.

6. M. Xu, H. Hao, L. Jiang, et al. "In vitro inhibitory effects of ethanol extract of Danshen (Salvia miltiorrhiza) and its components on the catalytic activity of soluble epoxide hydrolase," *Phytomedicine, vol.* 22, no. 4, pp. 444-451.
